# Downregulation of HIPK2 Increases Resistance of Bladder Cancer Cell to Cisplatin by Regulating Wip1

**DOI:** 10.1371/journal.pone.0098418

**Published:** 2014-05-20

**Authors:** Jun Lin, Qiang Zhang, Yi Lu, Wenrui Xue, Yue Xu, Yichen Zhu, Xiaopeng Hu

**Affiliations:** 1 Department of Urology, Beijing Friendship Hospital Affiliated to Capital Medical University, Beijing, P.R China; 2 Department of Urology, Beijing Chao-Yang Hospital Affiliated to Capital Medical University, Beijing, P.R China; German Cancer Research Center, Germany

## Abstract

Cisplatin-based combination chemotherapy regimen is a reasonable alternative to cystectomy in advanced/metastatic bladder cancer, but acquisition of cisplatin resistance is common in patients with bladder cancer. Previous studies showed that loss of homeodomain-interacting protein kinase-2 (HIPK2) contributes to cell proliferation and tumorigenesis. However, the role of HIPK2 in regulating chemoresistance of cancer cell is not fully understood. In the present study, we found that HIPK2 mRNA and protein levels are significantly decreased in cisplatin-resistant bladder cancer cell *in vivo* and *in vitro*. Downregulation of HIPK2 increases the cell viability in a dose- and time-dependent manner during cisplatin treatment, whereas overexpression of HIPK2 reduces the cell viability. HIPK2 overexpression partially overcomes cisplatin resistance in RT4-CisR cell. Furthermore, we showed that Wip1 (wild-type p53-induced phosphatase 1) expression is upregulated in RT4-CisR cell compared with RT4 cell, and HIPK2 negatively regulates Wip1 expression in bladder cancer cell. HIPK2 and Wip1 expression is also negatively correlated after cisplatin-based combination chemotherapy *in vivo*. Finally, we demonstrated that overexpression of HIPK2 sensitizes chemoresistant bladder cancer cell to cisplatin by regulating Wip1 expression.

**Conclusions:**

These data suggest that HIPK2/Wip1 signaling represents a novel pathway regulating chemoresistance, thus offering a new target for chemotherapy of bladder cancer.

## Introduction

Human bladder cancer is the tenth most common malignancy in women, and the fourth most common in men [1], [2]. Pathological studies indicate that bladder cancer comprises two major groups. The most common bladder cancer is urothelial carcinoma (UC) that usually recurs but rarely progress [3], [4]. In addition, invasive bladder cancer is more aggressive, and one-half of patients with invasive bladder cancer develop distant metastasis [5], [6]. Chemoradiation is a reasonable alternative to cystectomy in advanced/metastatic bladder cancer, but resistance to cancer chemotherapy is a common phenomenon especially in metastatic bladder cancer [7]. However, the advances in chemotherapy for the purpose of bladder cancer treatment have been limited because the underlying mechanisms causing chemoresistance are not known. Revealing the molecular mechanism of chemoresistance is indispensable for developing effective chemotherapeutic agents.

Homeodomain-interacting protein kinase-2 (HIPK2) is a serine/threonine kinase that as been shown to be involved in tumor suppressor [8], [9], [10]. HIPK2 is activated in response to various types of DNA-damaging agents, such as cisplatin, ultraviolet and roscovitine chemotherapeutic drugs [9]. HIPK2 phosphorylates p53 for specific activation of proapoptotic target genes, including p53AIP1, PIG3, Bax and Noxa and contributes to the regulation of p53-induced apoptosis [11], [12], [13]. Puca *et al* demonstrated that HIPK2 is an important regulator of p53 activity in response to a chemotherapeutic drug [14]. HIPK2 is expressed differently in sensitive versus chemoresistant cells in response to different chemotherapeutic drugs (i.e., cisplatin and adriamycin). HIPK2 inhibition suppresses the adriamycin-induced apoptosis in chemoresistant cancer cells, whereas overexpression of HIPK2 triggers apoptosis in chemoresistant cells, associated with induction of p53Ser46-target gene AIP1 [14], [15], [16]. Lazzari *et al* showed that HIPK2 knockdown induces resistance to different anticancer drugs even by targetingΔNp63α in p53-null cells [17].

Wild-type p53-induced phosphatase 1 (Wip1) is a p53-inducible serine/threonine phosphatase that switches off DNA damage checkpoint responses by the dephosphorylation of certain proteins, such as p38 mitogen-activated protein kinase, p53, checkpoint kinase 1 and checkpoint kinase 2 [18], [19]. Wip1 is targeted by HIPK2 for degradation [20]. Emerging data also indicate that Wip1 is overexpressed in various human tumors, and is associated with chemoresistance [19]. Wang *et al* showed that Wip1 knockdown increases DNA damage signaling and re-sensitizes oral squamous cell carcinoma (SCC) cells to cisplatin [21]. Using xenograft tumor models, they demonstrated that overexpression of Wip1 promotes tumorigenesis and its inhibition improves the tumor response to cisplatin [21]. Oppositely, Goloudina *et al* showed that Wip1 overexpression sensitizes colon cancer cells HCT116 (p53^−/−^) to cisplatin in RUNX2-dependent transcriptional induction of the proapoptotic Bax protein [22]. However, the role of Wip1 in regulating cisplatin sensitivity of bladder cancer cell is not fully understood.

Based on these findings, we investigated whether HIPK2 regulates chemosensitivity by targeting Wip1 in bladder cancer cell. Here we found that upregulation of HIPK2 inhibits Wip1 expression, which sensitizes chemoresistant bladder cancer cell to cisplatin.

## Materials and Methods

### Cell lines and tissue samples

The protocols used in the study were approved by the Hospital's Protection of Human Subjects Committee. Blood specimens were acquired with written informed consent from the Beijing Friendship Hospital Affiliated to Capital University of Medical Sciences. A total of 31 unresectable/metastatic bladder cancer patients were included in the study, and all the patients received cisplatin-based combination chemotherapy between 12/2011 and 08/2013 (median age 62.3, range 51–80).

Human bladder cancer cell lines with wild type of p53 (RT4 and 253J) were obtained and maintained as recommended by American Type Culture Collection (ATCC, Manassas, VA). The cisplatin-resistant subline RT4-resistance (RT4-CisR) was established by continuous exposure to increasing concentrations of cisplatin over a time period of 12 months, as reported previously [23].

### Real-time PCR

Total RNA was extracted from cells or tissues using Trizol reagent (Invitrogen, Carlsbad, CA), and reverse transcription (RT) reactions were performed according to the manufacturer's protocol. Real-time PCR was performed using a standard protocol from the SYBR Green PCR kit (Toyobo, Osaka, Japan). β-actin were used as references for mRNAs. ΔCt values were normalized to β-actin levels. The 2^–ΔΔCt^ method was used to determine the relative quantitation of gene expression levels. Each sample was analyzed in triplicate.

### Western blot analysis

Western blot analysis to assess HIPK2, Wip1 and β-actin expression was performed as previously described [24]. HIPK2 (ab28507) and Wip1 (ab72000) primary antibodies were purchased from Abcam (Cambridge, MA, USA). The β-actin primary antibodies were purchased from Sigma (MO, USA).

### Cell viability assay

Cells were plated and grown in 96-well plate in 0.1 ml Dulbecco's modified Eagle's medium containing 10% (v/v) fetal calf serum at 37°C for 24 h. Thereafter, the medium was changed and 0.1 ml fresh medium containing indicated drug was added and the cells were incubated for additional 48 h. The number of viable cells was determined by using the 3-(4,5-dimethylthiazol-2yl)-2,5-diphenyltetrazolium bromide (MTT) assay as described [25].

### RNAi and overexpression

RNAi was performed as described previously [26], [27]. The siRNAs used in this study were mixtures of three siRNAs and were purchased from Genepharm (Shanghai, China). pcDNA-HIPK2 and pcDNA-Wip1 were constructed to overexpress HIPK2 or Wip1 by introducing a fragment containing the HIPK2 or Wip1 precursor into pcDNA plasmid.

### Statistical analysis

All data are expressed as mean ± standard deviation (SD) from at least three separate experiments. The differences between groups were analyzed using Student's *t* test. Differences were deemed statistically significant at *p*<0.05.

## Results

### HIPK2 expression is decreased in chemo-resistant bladder cancer cell

**Figure 1:**
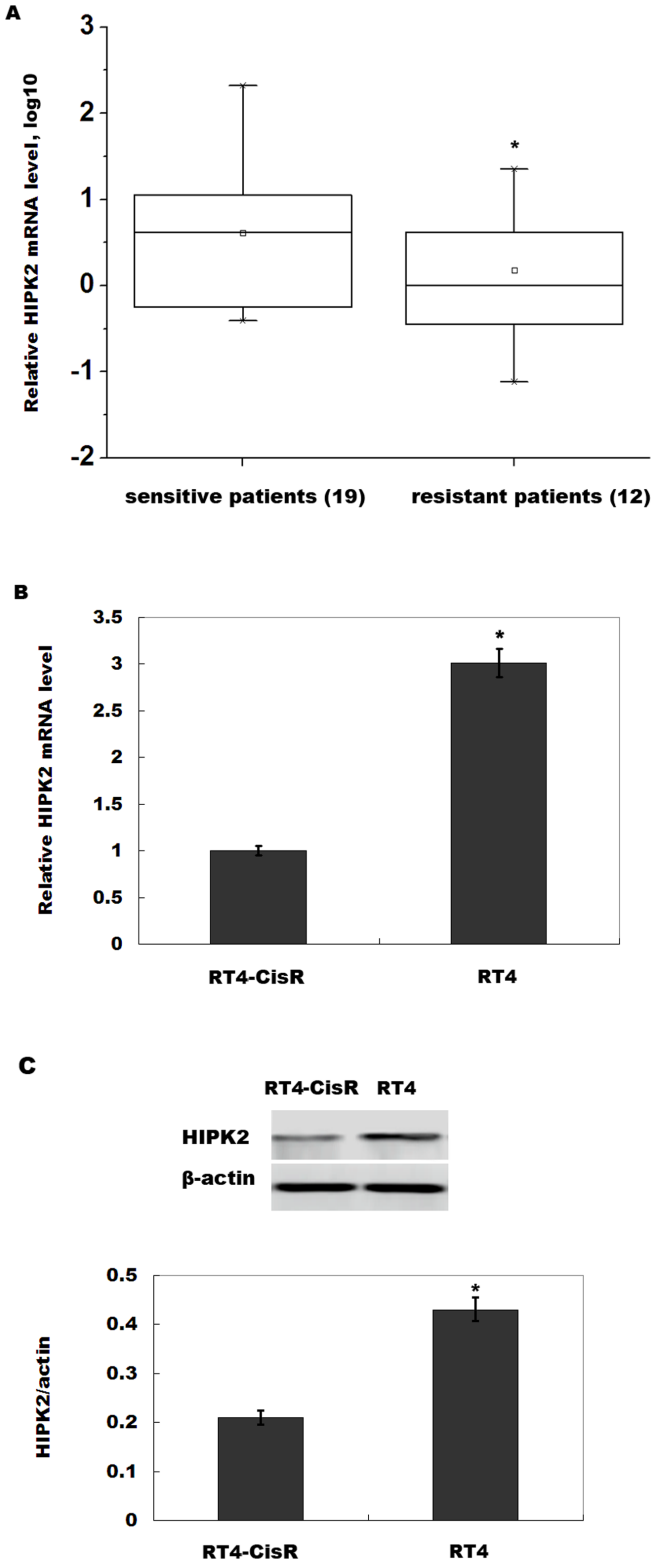
HIPK2 expression is decreased in chemo-resistant bladder cancer cell. (A) The analysis of the HIPK2 expression level was performed in blood samples with cisplatin-sensitive patients (n = 19) and cisplatin-resistant patients (n = 12). Total RNA was extracted and subjected to real-time RT-PCR to analyze the relative level of HIPK2 in each sample. Relative expression was calculated and normalized with respect to β-actin mRNA. All data were expressed as fold change relative to a tissue (control, expression = 1). The results were expressed as Log10 (2^−ΔΔCt^). *p<0.05. (B) The cisplatin-resistant subline RT4-CisR was established by continuous exposure to increasing concentrations of cisplatin over a time period of 12 months, and HIPK2 levels were analyzed by real-time PCR. Relative HIPK2 levels were calculated with respect to the control. *p<0.05. (C) Western blot analysis of HIPK2 protein level in RT4-CisR and RT4 cells (up). We also showed relative quantification of HIPK2 protein level (bottom, n = 3). *p<0.05.

Cisplatin is currently the most effective antitumor agent against advanced bladder cancer. However, resistance to cisplatin-based combination chemotherapy is a common phenomenon especially in metastatic bladder cancer. To clarify the molecular mechanisms underlying cisplatin resistance in bladder cancer, a total of 31 metastatic bladder cancer patients were included, and HIPK2 expression level was assayed after cisplatin-based combination chemotherapy. Figure 1A showed that HIPK2 expression in patients who are chemo-resistant is significantly decreased compared with chemo-sensitive patients. Then we established a cisplatin-resistant subline from the human bladder cancer cell line RT4 (RT4-CisR), and assayed the expression level of HIPK2. As shown in Figure 1B, HIPK2 mRNA levels were lower in RT4-CisR cells compared with RT4 cells. Similarly, HIPK2 protein levels were downregulated in RT4-CisR cells (Figure 1C). These data indicate that downregulation of HIPK2 may be related to cisplatin resistance of bladder cancer cells.

### HIPK2 knockdown increases cell viability during cisplatin treatment in bladder cancer cell

**Figure 2:**
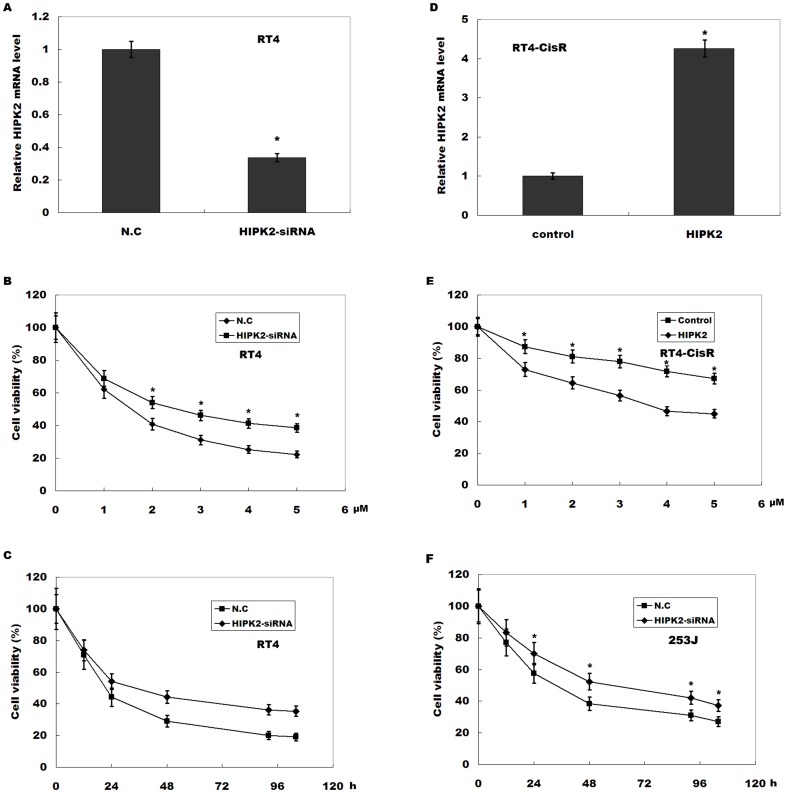
HIPK2 downregulation increases cell viability during cisplatin treatment in bladder cancer cell. (A) RT4 cells were transfected with HIPK2-siRNAs and HIPK2 expression level was assayed by real-time PCR. N.C = negative control (scrambled) siRNA. (B) RT4 cells were treated with HIPK2-siRNAs, and cell viability was assayed by using MTT following cisplatin treatment (1 to 6 µM). The results show data from six independent experiments, expressed as the mean ± SD. *p<0.05. (C) RT4 cells were treated with HIPK2-siRNAs, and at the indicated time points, cell viability was assayed by using MTT following cisplatin treatment (6 µM). The results show data from six independent experiments, expressed as the mean ± SD. *p<0.05. (D) RT4-CisR cells were transfected with pcDNA-HIPK2 and HIPK2 expression level was assayed by real-time PCR. (E) HIPK2 was overexpressed in RT4-CisR cells, and cell viability was assayed by using MTT following cisplatin treatment (1 to 6 µM). The results show data from six independent experiments, expressed as the mean ± SD. *p<0.05. (F) 253J cells were treated with HIPK2-siRNAs, and cell viability was assayed by using MTT following cisplatin treatment (6 µM). *p<0.05.

To investigate the role of HIPK2 in cisplatin resistance, separate overexpression and ablation experiments were done using either pcDNA-HIPK2 or HIPK2 siRNA during cisplatin treatment and cell viability was assayed. Figure 2A showed that HIPK2 expression levels were decreased in RT4 cells treated with HIPK2-siRNA. Then RT4 cell were incubated with different concentrations of cisplatin (0, 1, 2, 3, 4, 5 and 6 µM) for 48 h. As shown in Figure 2B, HIPK2 inhibition markedly increases RT4 cell viability compared with negative control (N.C). Expectedly, knockdown of HIPK2 increases RT4 cell viability following cisplatin treatment in time-dependent manner (Figure 2C). In RT4-CisR cells, cisplatin treatment resulted in a modest inhibition of cell viability, whereas overexpression of HIPK2 re-sensitized RT4-CisR cells to cisplatin (Figure 2D and E). Similarly, HIPK2 expression was inhibited in 253J cells after HIPK2-siRNA treatment (Figure S1), and HIPK2 inhibition increases 253J cell viability in time-dependent manner (Figure 2F). These data suggest that HIPK2 increases cisplatin sensitivity of bladder cancer cells.

### HIPK2 negatively regulates Wip1 expression

**Figure 3:**
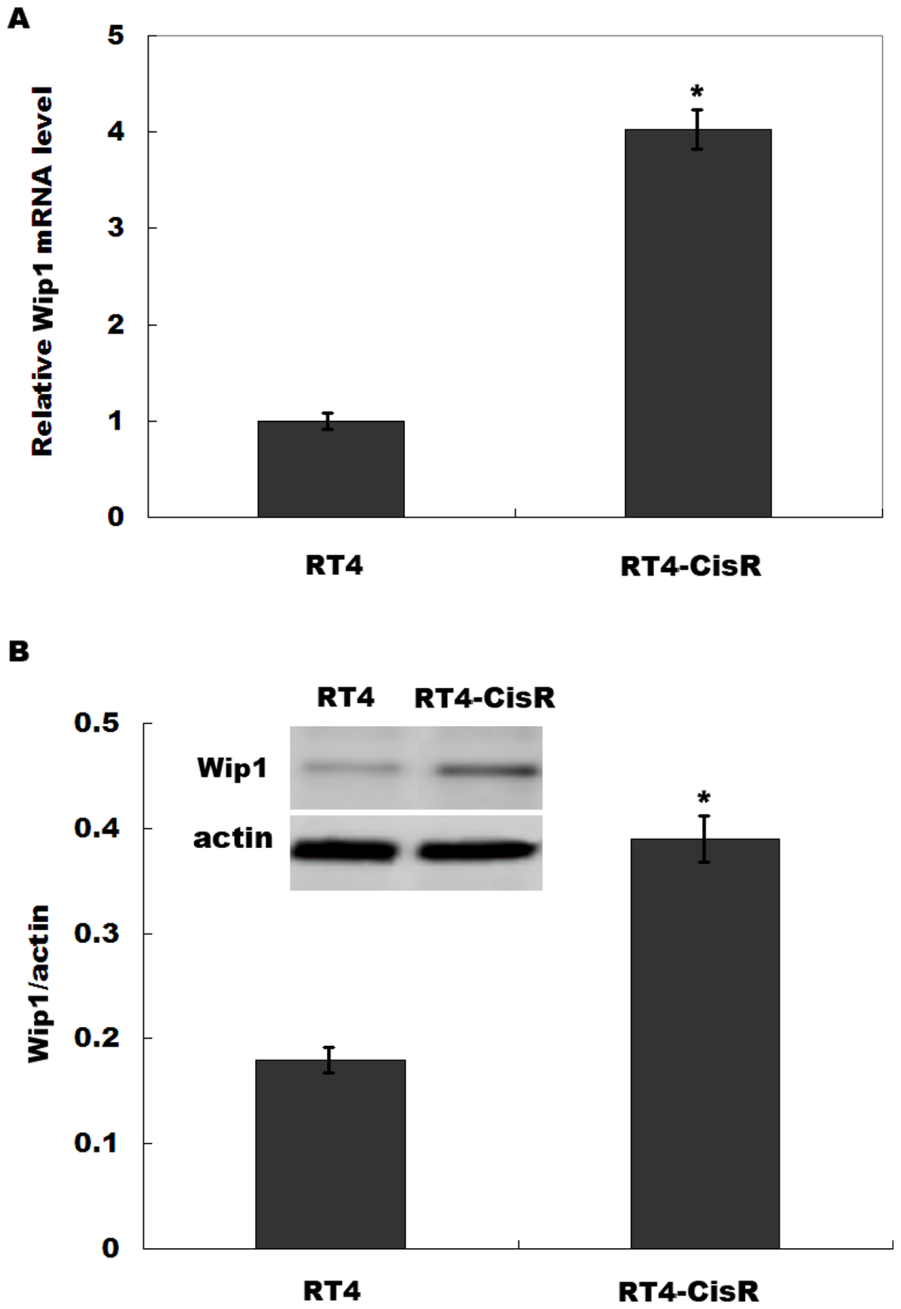
Wip1 expression is upregulated in RT4-CisR cell compared with RT4 cell. (A and B) Wip1 mRNA and protein expression levels were assayed in RT4 and RT4-CisR cells, respectively. *p<0.05.

**Figure 4:**
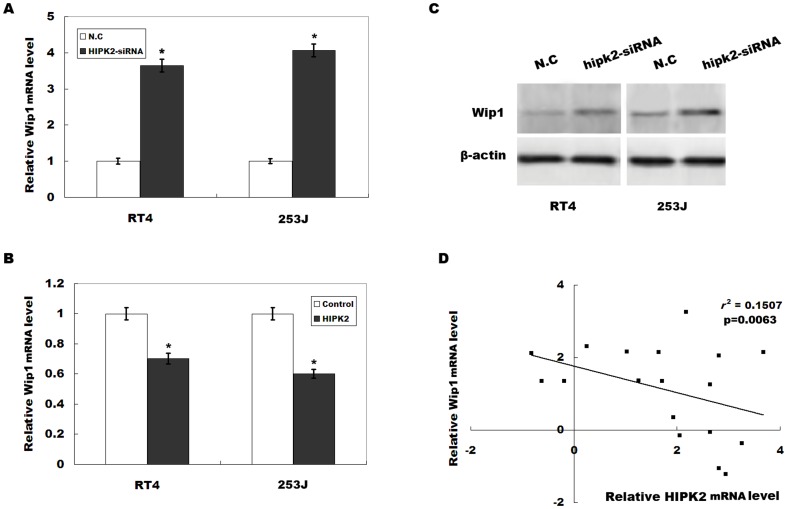
HIPK2 negatively regulates Wip1 expression. (A) Wip1 mRNA levels were evaluated by real-time PCR after HIPK2 inhibition in RT4 cells and 253J cells. *p<0.05. (B) Relative Wip1 mRNA level after HIPK2 overexpression in RT4 cells and 253J cells. *p<0.05. (C) Western blot analysis of Wip1 level after HIPK2 inhibition in RT4 and 253J cells. (D) Negative correlation between the HIPK2 levels and the Wip1 levels in 18 patients with bladder cancer after cisplatin-based combination chemotherapy (*r* ^2^ = 0.1507, *p* = 0.0063). Relative Wip1 or HIPK2 expression was calculated and normalized with respect to β-actin mRNA. All data were expressed as fold change relative to a tissue (control, expression = 1).

Previous studies showed that HIPK2 regulates tumor progression and drug resistance via several potential target genes, such as Bax, p53AIP1, Noxa, etc [14]. HIPK2 also plays a critical role in the initiation of double-strand break repair signaling by controlling Wip1 levels in response to ionizing radiation [20]. Recent studies indicate that Wip1 is overexpressed in various human tumors, and is associated with chemoresistance [19]. However, little is known about whether HIPK2 regulates cisplatin resistance by targeting Wip1. We first assayed the expression level of Wip1 in RT4 and RT4-CisR cells. Figure 3A and B showed that Wip1 mRNA and protein levels were significantly upregulated in RT4-CisR compared with RT4 cell. We then assayed whether HIPK2 negatively regulates Wip1 expression. HIPK2 knockdown increased Wip1 expression levels in bladder cancer cell lines (Figure 4A), whereas HIPK2 overexpression remarkably inhibited Wip1 mRNA level in bladder cancer cell lines (Figure 4B). Western blot analysis showed that HIPK2 knockdown increases Wip1 protein level (Figure 4C). *In vivo*, a significant negative correlation is also observed between the HIPK2 levels and the Wip1 levels in patients with bladder cancer after cisplatin-based combination chemotherapy (*r* ^2^ = 0.1507, *p* = 0.0063, Figure 4D). These data showed that downregulation of HIPK2 results in an increase of Wip1 expression.

### HIPK2 overexpression sensitizes chemoresistant bladder cancer cell to cisplatin by regulating Wip1 expression

**Figure 5:**
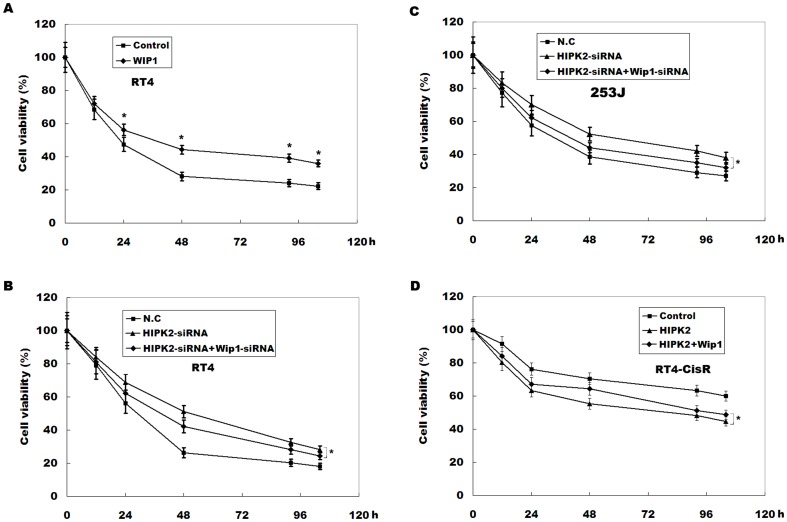
HIPK2 overexpression sensitizes chemoresistant bladder cancer cell to cisplatin by regulating Wip1 expression. (A) Wip1 was overexpressed in RT4 cells, and cell viability was assayed by using MTT following cisplatin treatment (6 µM). The results show data from six independent experiments, expressed as the mean ± SD. *p<0.05. (B and C) RT4 and 253J cells were treated with HIPK2-siRNA or HIPK2-siRNA plus Wip1-siRNA, and at the indicated time points, cell viability was assayed by using MTT following cisplatin treatment (6 µM). (D) HIPK2 or HIPK2 plus Wip1 was overexpressed in RT4-CisR cells, and cell viability was assayed by using MTT following cisplatin treatment (6 µM). *p<0.05.

We next investigated the role of Wip1 in regulating cell viability during cisplatin treatment. Figure 5A showed that Wip1 overexpression increased cell viability in RT4 cells during cisplatin treatment. HIPK2 inhibits Wip1 expression and decreases cisplatin resistance, and a significant negative correlation is observed between the HIPK2 and the Wip1. We therefore speculated that the role of HIPK2 in regulating cisplatin resistance is mediated by Wip1. Figure 5B showed that HIPK2 inhibition markedly increases RT4 cell viability compared with N.C, whereas Wip1 inhibition in HIPK2-downregulating cells partly reduces cell viability. Similarly, Wip1 inhibition in HIPK2-downregulating cells partly reduces 253J cell viability (Figure 5C). More important, cell viability is decreased by HIPK2 overexpression, whereas Wip1 overexpression increased HIPK2-overexpressing cell viability (Figure 5D). These data confirm that HIPK2 overexpression sensitizes chemoresistant bladder cancer cell to cisplatin by regulating Wip1 expression.

## Discussion

Human bladder cancer is one of the most fatal cancers all over the world, and its incidence is increasing in many countries. Besides surgical treatments, systematic chemotherapy, play an important role in bladder cancer treatment especially for patients with advanced and metastatic bladder cancer [28], [29]. However, despite a rapid shrinkage in tumor mass following chemotherapeutic cycles, the chemoresistance of cancer cells frequently results in the subsequent recurrence and metastasis of cancer [30], [31]. Considering the poor prognosis for patients with bladder cancer, mainly because of late diagnosis and low response to chemotherapy, we attempted to identify predictive markers of therapeutic response and molecular targets to increase sensitivity to treatment.

Our studies provide a rationale for the potential use of HIPK2 transduction to sensitize chemoresistant bladder cancer cells to cisplatin. We showed that HIPK2 expression levels are significantly downregulated in cisplatin-resistant RT4 cell (RT4-CisR) compared with RT4 cell. Downregulation of HIPK2 increases the cisplatin resistance in a dose- and time-dependent manner in RT4 cell, whereas forced expression of HIPK2 reduces the cell viability during cisplatin treatment. Moreover, overexpression of HIPK2 partially overcomes cisplatin resistance in RT4-CisR cell. Previous studies showed that HIPK2 is activated in response to various types of DNA-damaging agents, such as cisplatin, ultraviolet and roscovitine chemotherapeutic drugs [14], [32], and is an important regulator of p53 activity in response to a chemotherapeutic drug [11], [14]. Overexpression of HIPK2 in p53 wild-type re-sensitizes chemoresistant ovarian cancer cells to chemotherapy by mediating p53 phosphorylation. However, the molecular mechanism of HIPK2 in regulating chemoresistance of cancer cell is not fully understood.

Wip1 is a p53-inducible serine/threonine phosphatase that switches off DNA damage checkpoint responses by the dephosphorylation of certain proteins involved in DNA repair and the cell cycle checkpoint [19]. The Wip1 gene is amplified in many tumor types [33]. Song *et al* showed that Wip1 interacts with and dephosphorylates BAX to suppress BAX-mediated apoptosis in response to γ-irradiation in prostate cancer cells [19]. Radiation-resistant LNCaP cells showed dramatic increases in Wip1 levels and impaired BAX movement to the mitochondria after c-irradiation, and these effects were reverted by a Wip1 inhibitor [19]. Wang *et al* showed that Wip1 is an effective drug target for enhanced cancer therapy [21]. Wip1 inhibition increases DNA damage signaling and resensitizes oral SCC cells to cisplatin. Wip1 upregulation promotes tumorigenesis and its inhbition improves the tumor response to cisplatin. Consistent with above results, we found that expression level of Wip1 is upregulated in RT4-CisR cell compared with RT4 cell, and Wip1 overexpression increases cell viability during cisplatin treatment in RT4 cells. Importantly, we demonstrated that HIPK2 negatively regulates Wip1 expression in bladder cancer cell. HIPK2 and Wip1 expression is also negatively correlated after cisplatin-based combination chemotherapy *in vivo*. Forced expression of HIPK2 sensitizes chemoresistant bladder cancer cell to cisplatin by regulating Wip1 expression. **Conclusion**: These data demonstrated that HIPK2/Wip1 signaling represents a novel pathway regulating chemoresistance. Thus, this study reveals that HIPK2/Wip1 is an effective drug target for enhanced cancer therapy.

## Supporting Information

Figure S1 **HIPK2 expression level was assayed by real-time PCR in 253J cells.** N.C = negative control (scrambled) siRNA. *p<0.05.
(TIF)

## References

[1] Cohen SM, Shirai T, Steineck G (2000) Epidemiology and etiology of premalignant and malignant urothelial changes. Scand J Urol Nephrol Suppl: 105–115. 10.1080/00365590050509869 11144890

[2] Burger M, Catto JW, Dalbagni G, Grossman HB, Herr H, et al (2013) Epidemiology and risk factors of urothelial bladder cancer. Eur Urol 63: 234–241. 22877502 10.1016/j.eururo.2012.07.033

[3] Witjes JA, Comperat E, Cowan NC, De Santis M, Gakis G, et al (2014) EAU Guidelines on Muscle-invasive and Metastatic Bladder Cancer: Summary of the 2013 Guidelines. Eur Urol 65: 778–792. 24373477 10.1016/j.eururo.2013.11.046

[4] Kirkali Z, Chan T, Manoharan M, Algaba F, Busch C, et al (2005) Bladder cancer: epidemiology, staging and grading, and diagnosis. Urology 66: 4–34. 16399414 10.1016/j.urology.2005.07.062

[5] Pollack A, Zagars GK, Cole CJ, Dinney CP, Swanson DA, et al (1995) The relationship of local control to distant metastasis in muscle invasive bladder cancer. J Urol 154: 2059–2063 discussion 2063–2054 7500458

[6] Said N, Sanchez-Carbayo M, Smith SC, Theodorescu D (2012) RhoGDI2 suppresses lung metastasis in mice by reducing tumor versican expression and macrophage infiltration. J Clin Invest 122: 1503–1518. 22406535 10.1172/JCI61392 PMC3314474

[7] Chang JS, Lara PN Jr, Pan CX (2012) Progress in personalizing chemotherapy for bladder cancer. Adv Urol 2012: 364919. 22400017 10.1155/2012/364919 PMC3287014

[8] Wei G, Ku S, Ma GK, Saito S, Tang AA, et al (2007) HIPK2 represses beta-catenin-mediated transcription, epidermal stem cell expansion, and skin tumorigenesis. Proc Natl Acad Sci U S A 104: 13040–13045. 17666529 10.1073/pnas.0703213104 PMC1936219

[9] D'Orazi G, Rinaldo C, Soddu S (2012) Updates on HIPK2: a resourceful oncosuppressor for clearing cancer. J Exp Clin Cancer Res 31: 63. 22889244 10.1186/1756-9966-31-63 PMC3432601

[10] Hofmann TG, Glas C, Bitomsky N (2013) HIPK2: A tumour suppressor that controls DNA damage-induced cell fate and cytokinesis. Bioessays 35: 55–64. 23169233 10.1002/bies.201200060

[11] Puca R, Nardinocchi L, Givol D, D'Orazi G (2010) Regulation of p53 activity by HIPK2: molecular mechanisms and therapeutical implications in human cancer cells. Oncogene 29: 4378–4387. 20514025 10.1038/onc.2010.183

[12] D'Orazi G, Cecchinelli B, Bruno T, Manni I, Higashimoto Y, et al (2002) Homeodomain-interacting protein kinase-2 phosphorylates p53 at Ser 46 and mediates apoptosis. Nat Cell Biol 4: 11–19. 11780126 10.1038/ncb714

[13] Winter M, Sombroek D, Dauth I, Moehlenbrink J, Scheuermann K, et al (2008) Control of HIPK2 stability by ubiquitin ligase Siah-1 and checkpoint kinases ATM and ATR. Nat Cell Biol 10: 812–824. 18536714 10.1038/ncb1743

[14] Puca R, Nardinocchi L, Pistritto G, D'Orazi G (2008) Overexpression of HIPK2 circumvents the blockade of apoptosis in chemoresistant ovarian cancer cells. Gynecol Oncol 109: 403–410. 18395248 10.1016/j.ygyno.2008.02.018

[15] Hofmann TG, Moller A, Sirma H, Zentgraf H, Taya Y, et al (2002) Regulation of p53 activity by its interaction with homeodomain-interacting protein kinase-2. Nat Cell Biol 4: 1–10. 11740489 10.1038/ncb715

[16] Rinaldo C, Prodosmo A, Mancini F, Iacovelli S, Sacchi A, et al (2007) MDM2-regulated degradation of HIPK2 prevents p53Ser46 phosphorylation and DNA damage-induced apoptosis. Mol Cell 25: 739–750. 17349959 10.1016/j.molcel.2007.02.008

[17] Lazzari C, Prodosmo A, Siepi F, Rinaldo C, Galli F, et al (2011) HIPK2 phosphorylates DeltaNp63alpha and promotes its degradation in response to DNA damage. Oncogene 30: 4802–4813. 21602882 10.1038/onc.2011.182

[18] Takekawa M, Adachi M, Nakahata A, Nakayama I, Itoh F, et al (2000) p53-inducible wip1 phosphatase mediates a negative feedback regulation of p38 MAPK-p53 signaling in response to UV radiation. EMBO J 19: 6517–6526. 11101524 10.1093/emboj/19.23.6517 PMC305857

[19] Song JY, Ryu SH, Cho YM, Kim YS, Lee BM, et al (2013) Wip1 suppresses apoptotic cell death through direct dephosphorylation of BAX in response to gamma-radiation. Cell Death Dis 4: e744. 23907458 10.1038/cddis.2013.252 PMC3763429

[20] Choi DW, Na W, Kabir MH, Yi E, Kwon S, et al (2013) WIP1, a homeostatic regulator of the DNA damage response, is targeted by HIPK2 for phosphorylation and degradation. Mol Cell 51: 374–385. 23871434 10.1016/j.molcel.2013.06.010

[21] Wang L, Mosel AJ, Oakley GG, Peng A (2012) Deficient DNA damage signaling leads to chemoresistance to cisplatin in oral cancer. Mol Cancer Ther 11: 2401–2409. 22973056 10.1158/1535-7163.MCT-12-0448 PMC3496048

[22] Goloudina AR, Tanoue K, Hammann A, Fourmaux E, Le Guezennec X, et al (2012) Wip1 promotes RUNX2-dependent apoptosis in p53-negative tumors and protects normal tissues during treatment with anticancer agents. Proc Natl Acad Sci U S A 109: E68–75. 22065775 10.1073/pnas.1107017108 PMC3258624

[23] Esaki T, Nakano S, Masumoto N, Fujishima H, Niho Y (1996) Schedule-dependent reversion of acquired cisplatin resistance by 5-fluorouracil in a newly established cisplatin-resistant HST-1 human squamous carcinoma cell line. Int J Cancer 65: 479–484. 8621231 10.1002/(SICI)1097-0215(19960208)65:4<479::AID-IJC15>3.0.CO;2-5

[24] Xu N, Shen C, Luo Y, Xia L, Xue F, et al (2012) Upregulated miR-130a increases drug resistance by regulating RUNX3 and Wnt signaling in cisplatin-treated HCC cell. Biochem Biophys Res Commun 425: 468–472. 22846564 10.1016/j.bbrc.2012.07.127

[25] Wang F, Li X, Xie X, Zhao L, Chen W (2008) UCA1, a non-protein-coding RNA up-regulated in bladder carcinoma and embryo, influencing cell growth and promoting invasion. FEBS Lett 582: 1919–1927. 18501714 10.1016/j.febslet.2008.05.012

[26] Yang C, Li X, Wang Y, Zhao L, Chen W (2012) Long non-coding RNA UCA1 regulated cell cycle distribution via CREB through PI3-K dependent pathway in bladder carcinoma cells. Gene 496: 8–16. 22285928 10.1016/j.gene.2012.01.012

[27] Yuan G, Regel I, Lian F, Friedrich T, Hitkova I, et al (2013) WNT6 is a novel target gene of caveolin-1 promoting chemoresistance to epirubicin in human gastric cancer cells. Oncogene 32: 375–387. 22370641 10.1038/onc.2012.40

[28] Juffs HG, Moore MJ, Tannock IF (2002) The role of systemic chemotherapy in the management of muscle-invasive bladder cancer. Lancet Oncol 3: 738–747. 12473515 10.1016/s1470-2045(02)00930-0

[29] Gupta S, Mahipal A (2013) Role of systemic chemotherapy in urothelial urinary bladder cancer. Cancer Control 20: 200–210. 23811704 10.1177/107327481302000308

[30] Kamat AM, Sethi G, Aggarwal BB (2007) Curcumin potentiates the apoptotic effects of chemotherapeutic agents and cytokines through down-regulation of nuclear factor-kappaB and nuclear factor-kappaB-regulated gene products in IFN-alpha-sensitive and IFN-alpha-resistant human bladder cancer cells. Mol Cancer Ther 6: 1022–1030. 17363495 10.1158/1535-7163.MCT-06-0545

[31] Chung J, Kwak C, Jin RJ, Lee CH, Lee KH, et al (2004) Enhanced chemosensitivity of bladder cancer cells to cisplatin by suppression of clusterin in vitro. Cancer Lett 203: 155–161. 14732223 10.1016/j.canlet.2003.07.008

[32] Krieghoff-Henning E, Hofmann TG (2008) HIPK2 and cancer cell resistance to therapy. Future Oncol 4: 751–754. 19086839 10.2217/14796694.4.6.751

[33] Lu X, Nguyen TA, Moon SH, Darlington Y, Sommer M, et al (2008) The type 2C phosphatase Wip1: an oncogenic regulator of tumor suppressor and DNA damage response pathways. Cancer Metastasis Rev 27: 123–135. 18265945 10.1007/s10555-008-9127-x PMC2362138

