# Supplementary figures and images for: Downregulation of HIPK2 Increases Resistance of Bladder Cancer Cell to Cisplatin by Regulating Wip1
Source: PLoS One. 2014 May 20;9(5):e98418. doi: 10.1371/journal.pone.0098418 (PMC4028303; doi:10.1371/journal.pone.0098418)

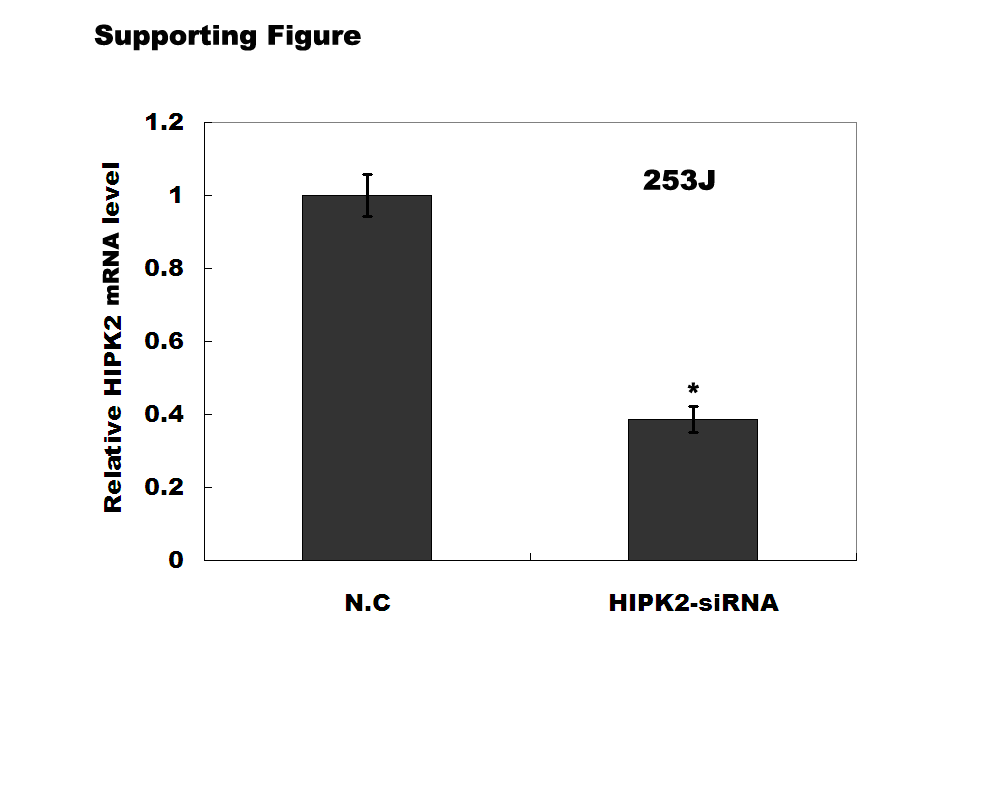

Supplement: Figure S1 — HIPK2 expression level was assayed by real-time PCR in 253J cells. N.C = negative control (scrambled) siRNA. *p<0.05. (TIF) [file pone.0098418.s001.tif]
